# A Necessary Reform in the Telephone Directory

**Published:** 1913-09-20

**Authors:** 


### A NECESSARY REFORM IN THE TELEPHONE DIRECTORY.

The Controller of the London Telephone Service has addressed a letter to the secretaries of hospitals stating that his attention "has been drawn to the fact that many persons have difficulty in turning up entries in the London Telephone Directory relating to hospitals." He goes on to suggest that as the difficulty usually has its origin in the fact that the exact title of the institution is not known by the person consulting the Directory, it would be advisable to place all these entries under the one heading of "Hospitals" instead of as at present.

There is no doubt that many hospitals have come to be known by some colloquial or abbreviated name quite dissimilar from their official title. It is doubtful, however, whether the remedy proposed by the Controller would be altogether effective, for unless the fact that all hospitals were grouped together was very plainly indicated in the Directory, members of the public might grope about to no purpose even more than they do at present. Then, again, if a person were looking for the institution known to nine people out of ten as Brompton Hospital, the entry "Hospital for Consumption, Fulham Road" (as it appears at present), would in many cases convey nothing. Furthermore, the existing difficulty is accentuated by the fact that the practice of giving official titles is not consistently adhered to. Thus the "National Hospital for the Paralysed and Epileptic" appears under "N," while the "National Hospital for Diseases of the Heart" appears under "H." To add to the complexity of the matter there is at least one London hospital of importance which has a telephone number but does not publish it. In view of these considerations it would seem that the best procedure, from the point of view of the public, would be to print the official title of each institution in its proper alphabetical position and then to add a list of those hospitals about whose titles there is likely to be any difficulty. This list could be printed at the beginning of the Directory and attention called to it under the heading of "Hospitals." No index or directory is complete which avoids cross references, and that is really the whole secret of this matter. Another aspect of importance, however, is seen in the article on "Telephones in Hospitals" which appears elsewhere in this issue.

